# The impact of 18F-FDOPA-PET/MRI image fusion in detecting liver metastasis in patients with neuroendocrine tumors of the gastrointestinal tract

**DOI:** 10.1186/s12880-020-00424-z

**Published:** 2020-02-24

**Authors:** O. Barachini, R. Bernt, S. Mirzaei, C. Pirich, K. Hergan, S. Zandieh

**Affiliations:** 1 Institute of Radiology and Nuclear Medicine, Hanusch-Hospital, Heinrich-Collin-Strasse 30, A-1140 Vienna, Austria; 2 Department of Nuclear Medicine with PET-Center, Wilhelminen-Hospital, Vienna, Austria; 3 Department of Nuclear Medicine, Paracelsus Medical University of Salzburg, Salzburg, Austria; 4 Department of Radiology, Paracelsus Medical University of Salzburg, Salzburg, Austria

**Keywords:** Magnetic resonance imaging, Neuroendocrine tumors, Liver, Metastasis, Positron emission tomography

## Abstract

**Background:**

This study assesses the value of image fusion using 18F-fluoro-L-DOPA (18F-DOPA) positron emission tomography (PET) and magnetic resonance imaging (MRI) for examining patients with neuroendocrine tumors (NETs) and a suspicion of metastasis of the liver.

**Methods:**

Eleven patients (five women and six men aged between 20 and 81, with a mean age of 54.6 years) were included in the study. All patients underwent whole-body 18F-DOPA PET examinations and contrast-enhanced MRI with diffusion-weighted sequences (DWS). Image fusion was performed using a semiautomatic voxel-based algorithm. Images obtained using PET and MRI were assessed separately. Side-by-side evaluations of fused PET/MRI images were also performed.

**Results:**

In total, 55 liver lesions (52 liver metastases and 3 benign lesions) were detected in the 11 patients. Sensitivity detection for liver lesions was higher when using PET/CT than when using contrast-enhanced MRI without DWSs and lower than using MRI with DWSs. The sensitivity of PET/MRI image fusion in the detection of liver metastasis was significantly higher than that of MRI with DWSs (*P* < 0.05).

**Conclusion:**

Images of the liver obtained using PET and MRI in patients with NETs exhibited characteristic features. These findings suggest that an appropriate combination of available imaging modalities can optimize patient evaluations.

## Background

Neuroendocrine neoplasms (NENs) are a heterogeneous group of tumors that arise in neuroendocrine cells dispersed throughout the body [1]. The main locations of origin for NENs are the gastroenteropancreatic (GEP) system and the lungs. Incidence rates for NENs that develop in the GEP (GEP-NENs) have increased significantly over the last 40 years [2], probably due to increased clinical awareness and improved diagnostic techniques. According to the database of the Surveillance, Epidemiology, and End Results Program, the estimated prevalence of GEP-NENs in the United States is 35 in 100,000 [3]. The Ki-67 protein is a cellular marker for proliferation that is present during all active phases of the cell cycle (G1, S, G2, and mitosis) and absent in resting cells (G0). The Ki-67 index often correlates with the clinical course of an NEN. According to World Health Organization classification, based on their Ki-67 index, NENs are divided into three grades (G1 ≤ 2%, G2 3–20%, G3 > 20%) and G3 NENs are distinguished from neuroendocrine carcinomas (NECs) by the level of histological differentiation, with NETs being well differentiated and NECs poorly differentiated.

Functional NENs associated with hormone excess syndromes, such as insulinomas, glucagonomas, gastrinomas, and vipomas, can be differentiated from nonfunctional NENs by the lack of hormonal symptoms; nevertheless, their hormonal production may be detected biochemically [4]—something which is true in the majority of cases. The high density of somatostatin receptors on the surface of NEN cells is a unique feature that is relevant for both diagnostic and therapeutic purposes [4].

Unfortunately, 40–50% of NEN patients present with distant metastases at the time of initial diagnosis, with the lymph nodes and liver the primary locations of said metastases [5]. The existence of distant metastases is associated with worse prognoses [6], with the extent of the hepatic tumor load being an important prognostic factor that influences therapeutic decisions [6]. Depending on tumor staging and grading, the therapeutic options for GEP-NENs include surgical resection (the only curative therapy) [7], somatostatin analogues, interferon, novel targeted drugs [8], chemotherapy [9], and peptide receptor radionuclide therapy with different radiolabelled somatostatin analogues (e.g., 177Lu-DOTA-octreotate) [10]. Liver metastases can be treated with surgical resection and ablative therapies (e.g., radiofrequency ablation, laser-induced thermotherapy, transarterial embolization, right portal vein embolization, and selective internal radiation therapy) [5].

Therapies related to treating the liver, particularly surgical and ablative methods, necessitate having reliable information about the number, size, location, and extent of liver metastases. Imaging methods that provide accurate information are therefore of the utmost importance. The most commonly used imaging modalities for the evaluation of liver metastases in NENs are contrast-enhanced computed tomography (CT), contrast-enhanced magnetic resonance imaging (MRI) with diffusion-weighted imaging (DWI), and positron emission tomography (PET) in combination with CT (PET/CT) and using various radiotracers.

Contrast-enhanced CT and MRI primarily depict morphology; an additional analysis of contrast enhancement dynamics can provide further insight into the microcirculation of hepatic tissue and metastases [11]. Previous studies have shown that DWI was more sensitive than contrast-enhanced MRI alone and that it identified additional NEN metastases [12, 13]. Depending on the radiotracer used, PET/CT can provide supplementary information about the receptor status and metabolism of the primary tumor and metastases.

The somatostatin analogues 68Ga-DOTATATE, 68Ga-DOTATOC, and 68Ga-DOTANOC can visualize well-differentiated NENs with generally low proliferation rates [14], while 18F-fluorodeoxyglucose (18F-FDG) is able to visualize poorly differentiated NENs with high proliferation rates. In clinical settings, however, there is a significant overlap in this regard.

18F-fluoro-L-DOPA (18F-DOPA) PET/CT shows increased DOPA decarboxylase activity in NENs. Since different types of malignant and nonmalignant lesions may show variable expression of somatostatin receptor (SSTR), it may be helpful to use 18F-DOPA as a tracer for catecholamine metabolic pathways when characterizing medullary thyroid cancer, midgut NENs, pheochromocytomas, neuroblastomas, and paragangliomas. The strength of the 18F-fluorodihydroxyphenylalanine (18F-FDOPA) radiotracer lies in its ability to detect well-differentiated and serotonin-secreting tumors in NENs [15–18].

Studies investigating PET/MRI fusion [19] and simultaneous PET/MRI [20–22] using 68Ga-DOTATOC as a tracer have shown promising results in terms of the detection and analysis of GEP-NENs and their metastases. The aim of the present study was to evaluate the practicability and potential of PET/MRI fusion including DWI, using 18F-FDOPA as a radiotracer.

## Methods

Liver MRI examinations were conducted using a 1.5-T machine (Achieva, Philips Medical System, Best, the Netherlands). The MRI sequences included the following: T1-weighted spoiled-gradient echo (SGE) with repetition time (TR) = 150 ms, echo time (TE) = 4.5 ms, 90° flip angle, 6 mm slice thickness (ST), 20% gap; and T1-weighted fat-suppressed SGE: TR = 175 ms, TE = 4 ms, 90° flip angle, 8 mm ST, 20% gap. This began 90–120 ms after the administration of gadolinium. The following sequences were then run: the T1-weighted out-of-phase SGE (TR = 135 ms, TE = 2.37 ms, 90° flip angle), the T2-weighted fat-suppressed turbo spin-echo (TR = 1000 ms, TE = 69 ms, 6 mm ST), and the T2-weighted fat-suppressed spin-echo (TR = infinite, TE = 90 ms, 18 sections acquired in 30 s of quiet breathing). A phased-array torso coil was used for the examination. A gadoterate meglumine contrast medium (Dotarem, Guerbet, USA) was administered in the form of a rapid bolus injection of 0.1 mmol/kg, followed by a normal saline flush. SGE image acquisition began immediately (45 s after the flush). Dynamic contrast material-enhanced imaging was performed at 20 s (arterial phase), 60 s (portal venous phase), and 120 s (equilibrium phase) after injecting gadoterate meglumine.

The parameters for DWI were as follows: TR/TE, 2000/63; b  = 600  s/mm^2^; FOV, 192  mm; matrix 128  ×  128 pixels; ST, 5  mm; intersection gap of 1  mm, with one signal acquired. The acquisition lasted approximately 40 min.

The study group consisted of 11 patients (five women and six men aged between 20 and 81, with a mean age of 54.6 years) (Table 1). All patients were examined with both MRI and 18F-DOPA PET/CT. For the PET examination, the patients fasted for a minimum of 4 h beforehand, but remained well hydrated orally, and 4 MBq of 18F-FDOPA per kilogram of bodyweight was administered intravenously. After an accumulation time of approximately 30 min, during which time the patient stayed in a sitting position, an emission scan from the base of the skull to the upper legs was performed.

**Table 1:** Characteristics of patients population including primary tumor, WHO grade and prior therapy

Primary tumor	WHO Grade	Therapy
Sigmoid colon	G2	no prior therapy
Bauhin valve	G2	no prior therapy
Ileum	G1	no prior therapy
Ileum	G1	no prior therapy
Ileum	G2	no prior therapy
Colon	G2	no prior therapy
Ileum	G2	no prior therapy
Ileum	G1	no prior therapy
Colon	G3	no prior therapy
Ileum	G2	no prior therapy
Ileum	G1	no prior therapy

A Philips Ingenuity TF (Philips Healthcare, PC Best, The Netherlands) PET/CT scanner with an axial field of view of 60 cm was used. Body scans were obtained, with each table position requiring a scanning time of 1.25 min. The images were reconstructed using a three-dimensional ordered-subsets iterative time-of-flight algorithm, after correction for scatter and attenuation. For the transmission scan (50 mA, 120 kV) prior to the emission scan, a collimation of 64 × 0.625 mm, an ST of 3 mm, and a reconstruction increment of 1.5 mm were used.

Whole-body acquisition lasted approximately 60 min for each patient. A fully automatic, multimodal image registration algorithm was used to fuse the images. For both modalities, landmarks used for image fusion included both the liver and the spleen. All the images were adjudged on a Philips workstation using commercial Philips IntelliSpace Portal software. The interval between the MRI and PET examinations was a maximum of 6 weeks.

Image analyses were conducted retrospectively by two experienced radiologists and two experienced nuclear medicine physicians, each of whom had more than 10 years’ experience. Kappa statistics were used to evaluate interobserver agreement. Kappa values of < 0.4, between 0.4 and 0.75, and > 0.75 were considered to represent poor, fair-to-good, and excellent agreement, respectively, based on the Fleiss classification [23].

On the MRI scans, metastasis was defined according to previously published criteria. On contrast-enhanced 18F-FDOPA PET/CT, metastases were identified either as: 18F-FDOPA-avid, when they showed higher 18F-FDOPA uptake than the normal adjacent liver and when they were seen on contrast-enhanced CT; or as 18F-FDOPA-non-avid when they were not seen on PET images but appeared on an IV contrast-enhanced CT [24].

Benign liver lesions were defined according to previously published characteristic parameters for MRI and CT results, respectively [25, 26]. A follow-up MRI was performed between 3 and 6 months later.

Biopsies was performed on one lesion per patient, and patients with multiple lesions exhibiting similar characteristics on imaging were presumed to have multifocal metastases of the same histologic type as that of the biopsy. The lesions were also verified in follow-up studies.

### Statistical analysis

Data analysis was performed using IBM SPSS for Windows Version 20 (IBM Corp., Armonk, NY, USA). Patient-based and lesion-based data analyses were performed. For quantitative analyses, ANOVA tests were performed to assess differences between the groups. *P* values < 0.05 were considered statistically significant.

## Results

The study found 55 liver lesions in 11 patients, with a mean of 6 ± 5 lesions per patient. Of the 11 patients, three exhibited more than 10 liver lesions. Of the 55 lesions, three (5.5%) were benign and had an average size of 10 ± 10 mm. In addition, 52 (94.5%) of the lesions were metastases and had a mean transaxial diameter of 14 mm (range: 4–41 mm; SD: 11.7 mm). Of these 52 liver metastases, 37 (71.1%) showed pathological 18F-DOPA uptake (Table 2).

**Table 2:** Characteristics of the liver lesions in patients with NET

Number of lesions	Mean Diameter	T2-Weighted	T1-Weighted	Dynamic- Sequence	DWI-Sequence	18F-DOPA-Uptake	Mean SUV	Diagnosis
52	14 mm	hyperintense 26(50%), hypointense 21(50%)	hypointense 52(100%)	positiv 29(55.7%)	positiv 46(88.4%)	positiv 37(71.1%)	6.6	metastasis
3	10 mm	hyperintense	hypointense	positiv	negativ	positiv	2.2	heamangioma

The positive predictive value (PPV) for 18F-FDOPA was 93%, while the negative predictive value (NPV) was 12%. The sensitivity for 18F-DOPA was 71% and the specificity was 40%. Dynamic contrast-enhanced magnetic resonance images (DCE-MRIs) showed that 29 (55.7%) of the liver metastases were positive, with only two of them in the arterial phase. In the DWI sequence, 46 (88.4%) of the liver metastases were positive. In total, 26 (50%) of the metastases were hyperintense on the T2-weighted sequence and hypointense on the T1-weighted sequence. Finally, 21 (40.3%) of the liver metastases were isointense on the T2-weighted sequence. The PPV for the DCE-MRI was 91% and the NPV was 88%. The sensitivity for the DCE-MRI was 91% and the specificity was 88%.

All of the metastases that remained undetected after using 18F-DOPA PET exhibited no elevated 18F-DOPA uptake and were less than 8 mm in size (8 ± 1 mm), as compared to metastases that were detected using 18F-DOPA PET, which were 20 ± 12 mm (*P* < 0.01). The maximum mean standardized uptake value (SUV) of all lesions was 6.6 (range: 0.9–18.7; SD: 3.9).

**Fig. 1:**
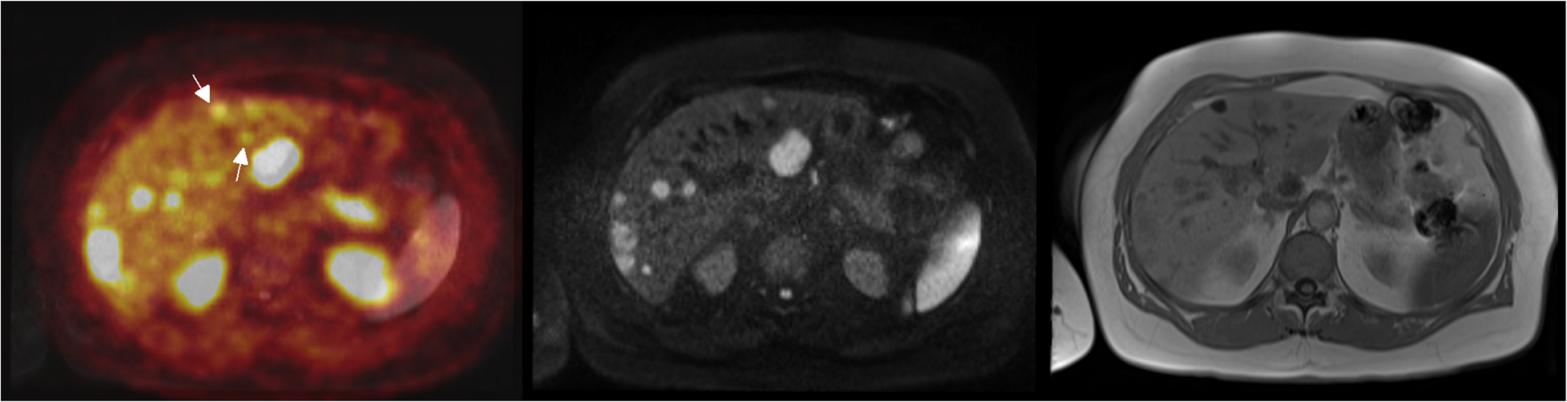
18F-FDOPA PET/MRI of a patient with primary neuroendocrine tumors shows multiple focal uptakes, suggestive of NET liver metastasis. PET and MRI mismatch was seen on the images

**Fig. 2:**
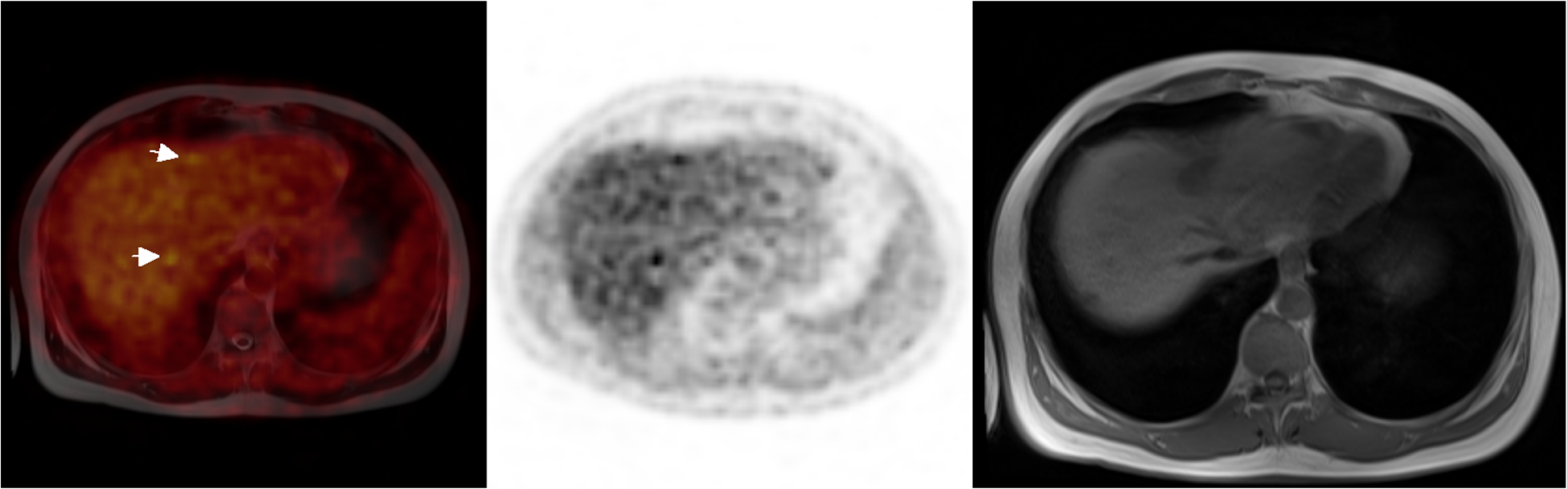
18F-FDOPA PET/MRI on the patient shows focal uptakes, suggestive of liver metastasis. The MRI/PET mismatch is depicted in this patient

Sensitivity was higher for PET/CT than for contrast-enhanced MRI without DWI; it was lower for contrast-enhanced MRI with DWI. Using DWS for both readers, the sensitivity of PET/MRI image fusion in detecting liver metastases was significantly higher than that of PET/CT and MRI (*P* < 0.05; Figs. 1 and 2). For PET/MRI image fusion, the sensitivity was 96% and the specificity was 88%. The PPV for PET/MRI image fusion was 95% and the NPV was 88%. Interobserver agreement between the two readers was excellent (kappa value = 0.80).

## Discussion

Our study shows that an appropriate combination of available imaging modalities can optimize patient evaluations. The sensitivity of the PET/MRI image fusion demonstrates that liver metastasis detection is significantly higher when using that technique as opposed to MRI with DWS. The 18F-FDOPA PET tracer provides additional metabolic and functional information that gives valuable additional diagnostic data on patients with NETs [16].

Corrias et al. reported imaging features of malignant abdominal neuroendocrine tumors with rare presentation in four patients [27]. The location and type of NENs could influence the sensitivity of the diagnostic methods. 18F-DOPA PET/CT has very high sensitivity to midgut NENs but low sensitivity for foregut NENs. One explanation for the high specificity of 18F-DOPA PET/CT in patients with NENs may be that only neuroendocrine cells can take up, decarboxylate, and store amino acids and their amines. 18F-DOPA is not taken up in significant proportions by inflammatory cells, which is an important advantage over other PET tracers, such as gallium-68-somatostatin analogues and fluorine-18-fluorodeoxyglucose.

Infectious or inflammatory processes may also be incidentally detected on PET/CT examination. There is no reliable SUV threshold that can routinely differentiate infection or inflammation related to malignancy from infectious/inflammatory processes that are hypermetabolic or other non-FDG avid malignancies, such as cystic or necrotic neoplasms [28].

Besides 18F-DOPA PET/CT, the most widely used PET technique for NEN imaging is SSTR PET/CT using gallium-68-somatostatin analogues (i.e., DOTA-NOC, DOTA-TOC, or DOTA-TATE). Recent meta-analysis shows high diagnostic accuracy in this setting [29, 30]. The use of F-18-FDG-PET/CT is usually limited to patients with high G2 or G3 tumors. Some centers perform PET/CT with both 68Ga-DOTA-SSA and FDG in all patients that have G2 or G3 tumors, because of the prognostic information that is provided when tracking FDG positivity or negativity in tumors.

The different functional characteristics of neuroendocrine cells could also be depicted using 18F-DOPA and gallium-68-somatostatin analogues. The selection for well-differentiated NENs should be guided by the biology of the NENs. Features specific to NENs, such as taking up and decarboxylating l-DOPA and transforming it into dopamine, make 18F-DOPA suitable for depicting NENs with elevated serotonin levels [31, 32].

The receptor-based uptake mechanism in gallium-68-somatostatin analogues allows NEN lesions to be depicted independently of their functional activity. Patients could be selected prior to peptide receptor radionuclide therapy by using gallium-68-somatostatin analogues.

The overall superiority of SSR PET/CT compared to 18F-DOPA is evidenced in the few available studies that compare SSR and 18F-DOPA PET/CT in patients with GEP and thoracic NENs. Future comparison studies taking into account the different locations of GEP and thoracic NENs will be necessary to confirm the superiority of SSR PET/CT over 18F-DOPA.

To our knowledge, there have been no significant data comparing SSR and 18F-DOPA PET/CT in patients with Pheochromocytoma and paraganglioma. A study comparing SSR and 18F-DOPA PET/CT in patients with recurrent medullary thyroid cancer illustrated the superiority of 18F-DOPA over SSR PET/CT. More comparison studies are required to establish guidelines for the choice of PET radiopharmaceuticals for evaluating NENs in clinical practice.

Moryoussef et al. conducted a retrospective analysis of 25 abnormal livers and 22 abnormal whole-body standard MRIs [12]. They reported that the addition of DWI sequences to standard liver MRI sequences yielded additional findings in 45% of patients, with the detection of 1.78 times more new lesions. The same study showed that this resulted in a management change for 18% of the patients.

Armbruster et al. conducted a DCE-MRI and PET/CT study of 32 patients with NENs and hepatic metastases, using either 18F-FDG or 68Ga-DOTATATE as tracers [33]. They demonstrated that both PET/CT parameters and dynamic contrast-enhanced (DCE)-MRI perfusion parameters showed high diagnostic accuracy in distinguishing between liver metastases and liver tissue. They suggested that the two modalities provided complementary information.

DCE-MRI is currently the state-of-the-art imaging method used for the detection and characterization of liver lesions. High b-values (≥ b100) produced an increased contrast between the background liver and lesions and supported the detection of focal liver lesions [34]. While DWI alone is less sensitive than a gadoxetic acid-enhanced MRI in detecting liver metastases, it increases the sensitivity of detection for liver metastases to 90.6–95.5%, when combined with DCE-MRI [35].

Contrast-enhanced MDCT features correlate with histological findings and enable differentiation between G1 and G2 pNETs during preoperative examinations [36]. Third generation DECT, with an assessment of iodine uptake, improves the differentiation of hepatic NET metastasis and hepatocellular carcinoma (HCC) in noncirrhotic livers, as shown in a study by Kaltenbach et al. that examined 46 patients with either hepatic NEN metastasis or HCC, all of whom had undergone liver DECT in a retrospective study [37].

In the present study, we investigated the value of image fusion with 18F-DOPA and MRI in patients with NETs and a suspicion of metastasis of the liver. The results were significant when comparing the detection rate of liver metastasis using PET/MRI image fusion and PET/CT or MRI with DWSs (*P* < 0.05). Gaspard et al. reported that DW MRI was more sensitive than fat-suppressed T2-weighted fast spin echo (FSE) and gadolinium-enhanced T1-weighted imaging, and that a combination of sequences improved the detection of liver metastases [13]. In their study, the best results were obtained by combining DW, fat-suppressed T2-weighted FSE, and gadolinium-enhanced T1-weighted sequences, and they detected a total of 162 liver metastases in 41 patients. The findings of the present study were in accordance with the results of the study by Gaspard et al. A novel aspect of the present study was the use of additional information provided by 18F-FDOPA PET, something that has not been reported previously.

The use of diagnostic imaging continues to expand and is routinely employed in clinical settings. The present study demonstrates the potential role that 18F-FDOPA PET/MRI fusion could play in detecting liver metastasis in patients with NETs of the gastrointestinal tract. A limitation of the present study was the delay (a maximum of 6 weeks) between the MRI and PET examinations. This delay may have had a partial response effect on the metabolic activity. However, as shown in Table 1, there was still enhanced uptake, indicating disease activity in these areas. Our data show that 18F-FDOPA PET/MRI fusion imaging may be useful in the diagnosis of liver metastasis in patients, however, the small patient population and the retrospective nature of the study do not allow us to draw any detailed conclusions. Larger studies with long-term follow-up are needed to confirm the effectiveness of the hybrid approach.

Overall, the findings of the present study confirm that 18F-FDOPA PET-MRI is clinically useful for the detection of NET liver metastasis.

## Conclusion

The fusion of 18F-FDOPA-PET and MRI allows clinicians to obtain a morphofunctional map in patients with NET. The data show that 18F-FDOPA PET/MRI fusion imaging may be useful in the diagnosis of liver metastasis in patients with NETs of the gastrointestinal tract.

## Data Availability

Data to replicate findings are presented in the tables of the main paper. Due to patient privacy protection, any additional materials from the study are available only upon individual request directed to the corresponding author.

## Abbreviations

18 F-FDG-PET: 18 F-fluor-desoxyglucose positron emission tomography
CT: Computed tomography
DWSs: Diffusion-weighted sequences
MRI: Magnetic resonance imaging
NETs: Neuroendocrine tumors
